# Agreement and reproducibility between 3DStent vs. Optical Coherence Tomography for evaluation of stent area and diameter

**DOI:** 10.1007/s10554-024-03268-8

**Published:** 2024-10-28

**Authors:** Andrea Ruberti, Riccardo Rinaldi, Giovanni Occhipinti, Liliane Ramus, Giulio Guagliumi, Manel Sabate, Salvatore Brugaletta

**Affiliations:** 1 Cardiovascular Clinic Institute, Institut d’Investigacions Biomèdiques August Pi i Sunyer (IDIBAPS), Hospital Clínic, University of Barcelona, c/ Villarroel 170, Barcelona, 08036 Spain; 2 GE HealthCare, Buc, France; 3 Galeazzi-Sant’Ambrogio Hospital IRCCS, Milan, Italy; 4 Present Address: Facultat de Medicina i Ciències de la Salut, Universitat de Barcelona (UB), 08036 Barcelona, Spain

**Keywords:** 3DStent, OCT, Stent optimization, Imaging

## Abstract

3DStent is a novel rotational angiography imaging capable of 3D reconstruction and measuring stent area and diameter, without need for intravascular imaging. To compare 3DStent and OCT-derived stent area and diameter after PCI. Patients with de novo coronary lesions who underwent treatment with a single DES and evaluated by OCT and 3DStent were included. Stent area and diameter were measured by 3DStent, at abluminal, mid and endoluminal side and by OCT. From September 2023 to February 2024 six coronary lesions were analyzed. Post-PCI stent area measured by OCT was (mean ± standard deviation) 7.03 ± 2.85 mm^2^ and by 3DStent 9.41 ± 2.79 mm^2^, 7.21 ± 2.23 mm^2^ and 5.63 ± 1.83 mm^2^ at abluminal, mid and endoluminal side, respectively. Stent diameter by OCT was 2.93 ± 0.58 mm, and by 3DStent 3.27 ± 0.50 mm, 2.86 ± 0.49 mm and 2.52 ± 0.45 mm at abluminal, mid and endoluminal side, respectively. Significant correlation was observed between OCT and 3DStent in relation to stent area (Exp(B) 3.35, mean of difference 0.19 ± 1.01 mm^2^, 95%CI -1.80–2.17 mm^2^, *p* < 0.001) and diameter (Exp(B) 3.18, mean difference − 0.07 ± 0.18 mm, 95%CI -0.43–0.30 mm, *p* < 0.001), particularly when 3DStent measurements performed at the mid side. Very high reproducibility was demonstrated by intra- and inter-observer analysis (*r* = 0.92 and *r* = 0.93 respectively). 3DStent appears to be an easy and reproducible tool to assess post-PCI stent area and diameter as compared to OCT.

## Introduction

Intravascular imaging (IVI)-guided percutaneous coronary intervention (PCI) has been associated with reduction in cardiac death, stent thrombosis, and target lesion revascularization compared with angiography-guided PCI [1]. Optimal stent expansion, achieved by maximizing minimal stent area (MSA), is independently associated with superior long-term clinical outcomes, providing a partial explanation for the observed clinical benefits of IVI [2–5]. Specifically, a minimum stent area (MSA) > 4.5mm^2^ by optical coherence tomography (OCT) is usually considered the threshold related to good long-term results [6–9]. Nevertheless, significant barriers exist against the routine use of IVI-guided PCI related to procedural time, costs and in case of OCT injection of additional contrast [9–11]. 

3DStent (GE HealthCare, Chicago, IL) is a novel rotational non-injected angiography-based imaging that generates a 3D multi-planar reconstruction (MPR) of the stent, enabling assessment of stent area and diameter, without the need for IVI or additional contrast.

There are no data comparing 3DStent vs. OCT. We report the first-in-man analysis of agreement and reproducibility of 3DStent vs. OCT-based stent area/diameter assessment.

## Methods

### Study design and population

This is an investigator-initiated, single-center, retrospective study performed at the Hospital Clinic Barcelona (Barcelona, Spain). All consecutive ≥18year-old patients presenting symptomatic coronary artery disease (CAD) with de novo lesions (type A, B1 and B2) [12] in vessels > 2.5 mm, who underwent PCI with implantation of a single DES and subsequent assessment by both OCT and 3DStent were included. PCI was performed according to standard clinical practice. All participants in this study provided written informed consent for percutaneous coronary intervention. The present study was approved by the local ethics committee.

### 3DStent and OCT acquisition

#### 3DStent

The Allia IGS 7 with AutoRight™ (GE Healthcare, Chicago, IL, USA) was used for 3DStent acquisition. 3DStent reconstruction is performed using C-arm Motion Compensated Computed Tomography (CMCT) resulting in an intraprocedural 3D visualization. The patient lays in supine position with one arm above the head in order to optimize the 3DStent image quality. 3DStent relies on a 200 degrees rotational angiography. The acquisition workflow is largely automated: the operator centers the stent in 2 angulations and verifies during an X-ray–free test that the rotation is collision-free. After an automatic test spin, 3DStent rotational cine acquisition is performed with an automatic synchronization of X-ray exposure, gantry rotation and it can be launched from the control room with no impact on the operator dose. During the acquisition, similarly to digital stent enhancement, a deflated balloon must be kept inside the stent to allow stent localization by the software. 3DStent acquisition has two possible rotation speeds: 10°/s and 20°/s. The radiation dose is lower (half dose) when 20°/s rotation is used, however 10°/s rotation offers higher image quality: in our study 10°/s rotation speed was used.

3DStent software - with an axial and longitudinal resolution of 100 μm - automatically generates a 3D model and multiplanar reconstruction from the acquired frames, enabling a multislice cross-sectional evaluation of the stent architecture.

#### OCT

Standard OCT imaging was performed using Dragonfly™ OPTIS™ imaging catheter (Abbott Vascular, Lake County, IL, USA) after stent implantation. The OCT acquisition was performed using a commercially available system for intracoronary imaging (LightLab Imaging Inc, Westford, MA, USA). Automated pull-back at 25 mm/s was performed in concordance with blood clearance by the contrast injection [9]. 

Both 3DStent or OCT were performed at the same stage of the PCI procedure in order to allow for a direct comparison between the two techniques in terms of stent diameter and area.

### Imaging analysis

**Fig. 1:**
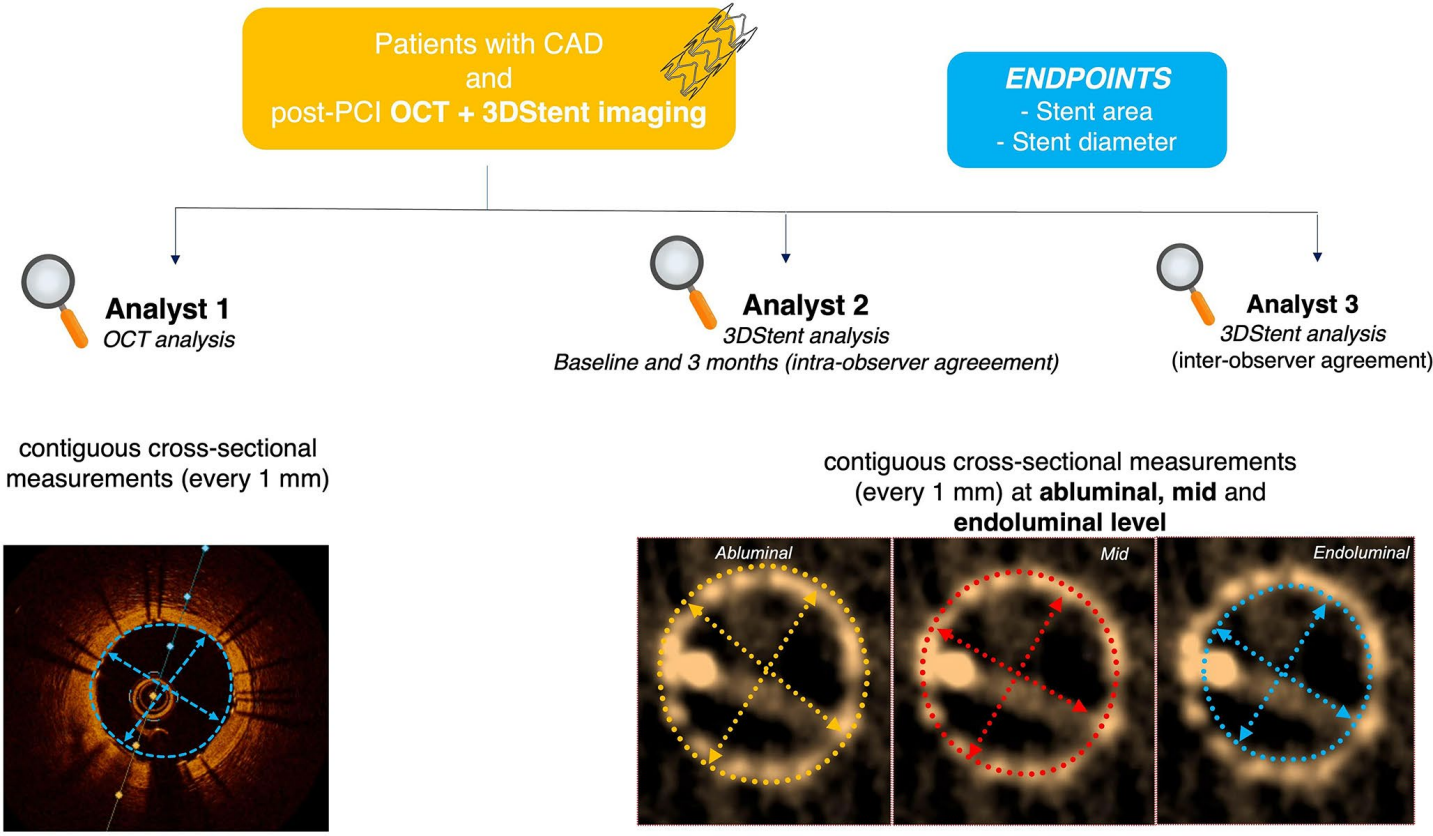
Study design. CAD = Coronary Artery Disease; OCT = Optical Coherence Tomography; PCI = Percutaneous Coronary Intervention

Two interventional cardiologists independently performed offline 3DStent and OCT analysis, blinded to the alternative technique. Stent area and diameter were assessed at contiguous cross-sections every 1 mm in both modalities in order to obtain comparable cross sections in 3DStent and OCT pullbacks. (Fig. 1)

3DStent reconstructions were analyzed using the GE HealthCare Advantage review workstation of cathlab, which allows measurement of stent area and diameter at each frame (10 frames per mm). To the aim of this analysis, one cross section was selected at every 1 mm [13, 14]. Unlike OCT, which has clear guidelines for stent measurements, there are currently no guidelines about stent area contouring with the 3DStent: therefore, per each 3DStent cross-section analyzed, we measured stent area and diameters by delineating three different contours at the abluminal, mid, and endoluminal side of the stent. (Fig. 1)

OCT measurements of stent area and diameter were performed as usual [9]: offline OCT data analysis was carried out using specific proprietary software for off-line analysis (LightLab Imaging Inc, Westford, MA, USA) and software-enabled automatic strut detection with manual corrections allowed only in case of huge anomalies.

### Intra-observer and inter-observer agreement for 3DStent

For the intra-observer agreement, the same analyst repeated the measurements on the same 3DStent reconstruction 3 months later, by using the same methodology described above. For the inter-observer agreement, a third analyst – with the same experience than the other two - was involved to perform the 3DStent analysis on the same cross-sections analyzed by the first analyst, by using the same methodology.

### Statistical analysis

Continuous variables are presented as mean ± standard deviation (SD), while categorical variables are represented as numbers and frequencies. 3DStent and OCT measurements were analyzed at the cross-sectional level using multilevel adjusted General Estimating Equations (GEE). Agreement between 3DStent and OCT analysis of stent area and diameter was also established by the Bland-Altman test.

Intra-observer and inter-observer agreements regarding stent area and diameter by 3DStent were assessed using the Spearman test for correlation. Regarding the intra- and inter-observer agreement, Spearman r value of 0.01–0.19 indicates no or negligible agreement, 0.20–0.29 indicates weak agreement, 0.30–0.39 indicates moderate agreement, 0.40–0.69 indicates strong agreement, ≥ 0.79 indicates very strong agreement [15, 16]. 

A two-tailed p-value ≤ 0.05 was deemed statistically significant. Statistical analyses were conducted using IBM SPSS 20.0 Statistics software (SPSS Inc., Chicago, IL, USA).

## Results

### Patient population

**Fig. 2:**
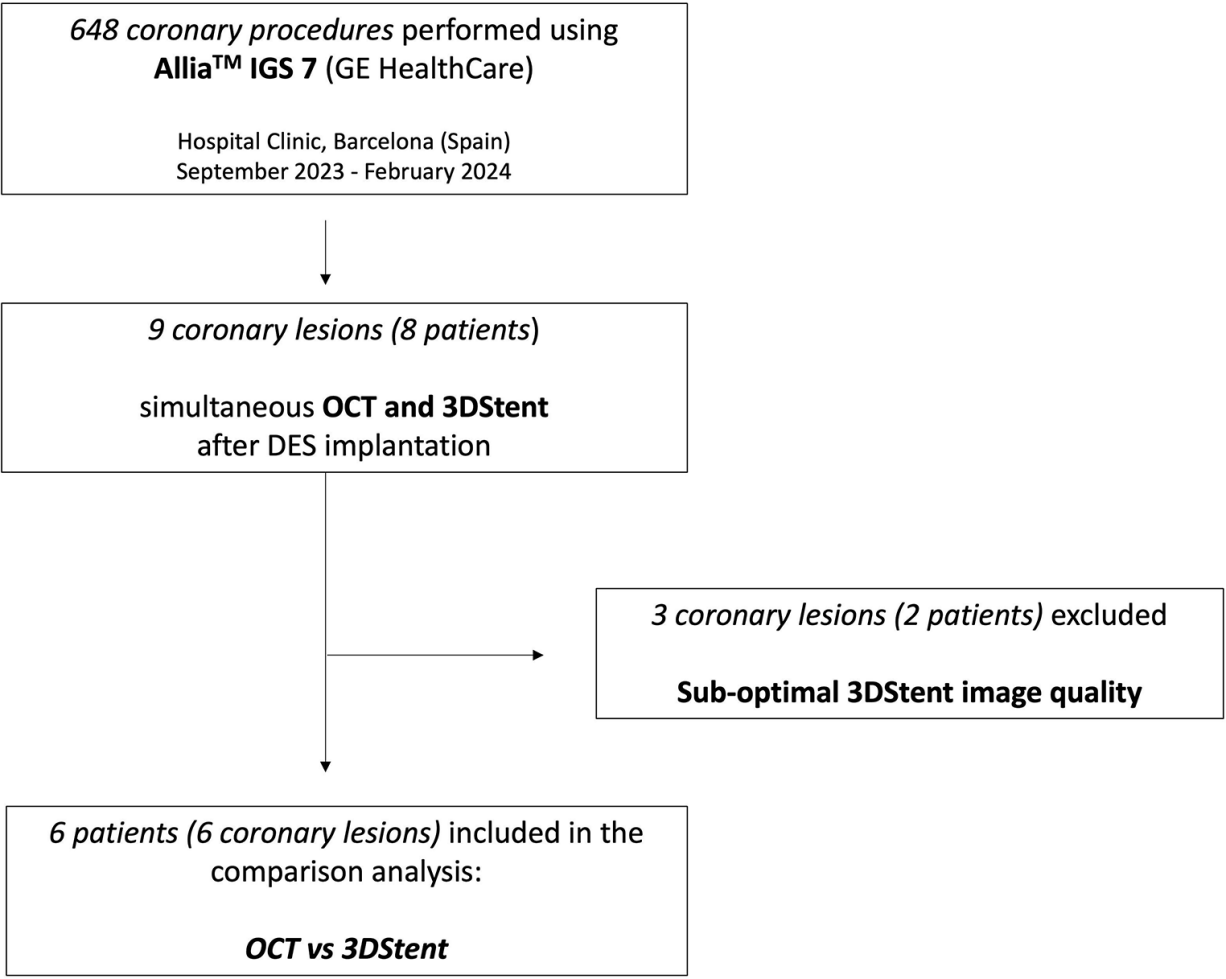
Flowchart of the study. DES = Drug-Eluting Stent; OCT = Optical Coherence Tomography; PCI = Percutaneous Coronary Intervention

Between September 2023 and February 2024, a total of 648 coronary procedures were performed by using the Allia™ IGS 7 cathlab with 3DStent (GE HealthCare, Chicago, IL, USA). Out of them, 76 (11%) were PCI with DES implantation; 3DStent acquisition was obtained in 10 (13%) patients and 11 coronary lesions, whereas OCT in 10 (13%) patients with 10 lesions. Both modalities were used in 8 (10%) patients with 9 coronary lesions. Out of them, 2 patients (25%) and 3 coronary lesions (33%) were excluded due to sub-optimal imaging quality of 3DStent: among the excluded cases, one lesion had motion artifacts during rotational angiography, leading to some artifacts in the 3D reconstruction, whereas two patients had a BMI > 30 kg/m^2^ and one patient had also COPD. Eventually, 6 patients with 6 coronary lesions were included in our analysis (Fig. 2).

Mean age of the analyzed cohort was 68.3 ± 7.8 years, 75% were males. Complete demographic and procedural data are exposed in Tables 1 and 2.

**Table 1:** Baseline clinical characteristics

Clinical characteristics	Population (*n* = 6)
Age (y)	68.3 ± 7.8
Sex (female), n (%)	2/6 (33.3)
BMI (Kg/m^2^)	28.4 ± 4.8
Diabetes, n (%)	3/6 (50)
Family history of coronary artery disease, n (%)	2/6 (33.3)
Arterial Hypertension, n (%)	6/6 (100)
Dyslipidemia, n (%)	6/6 (100)
Smoking, n (%) - no - current - former	− 3/6 (50) − 1/6 (16.7) − 2/6 (33.3)
Chronic kidney disease (eGFR < 60 ml/min/m^2^), n (%)	0/6 (0)
Chronic obstructive pulmonary disease, n (%)	1/6 (16.7)
Previous myocardial infarction, n (%)	0/6 (0)
Previuous PCI, n (%)	0/6 (0)
Previous coronary artery bypass, n (%)	0/6 (0)
Clinical presentation, n (%)	
- CCS - UA/NSTEMI	− 5/6 (83.3) − 1/6 (16.3)

BMI = Body Mass Index; CCS = Chronic Coronary Syndrome; NSTEMI = Non-ST-Elevation Myocardial Infarction; PCI = Percutaneous Coronary Intervention; UA = Unstable Angina Pectoris

**Table 2:** Procedural data

Procedural data	Coronary artery (*n* = 6)
Vessel (%)	
- LAD - LCX - RCA	1/6 (16.7) 3/6 (50) 2/6 (33.3)
Radial access (%)	6/6 (100)
Contrast media (ml)	134.2 ± 36.6
Total Dose-Area Product Radiation (Gy.cm^2^)	104.0 ± 30.4
Postdilatation (%)	5/6 (83.3)
Mean stent diameter (mm)	3.0 ± 0.3
Mean stent length (mm)	24.5 ± 9.2
**OCT data**	
Mean stent Area (mm^2^)	7.03 ± 2.85
Mean stent Diameter (mm)	2.93 ± 0.58
**3DStent data**	
Mean stent area abluminal (mm^2^)	9.41 ± 2.79
Mean stent area mid (mm^2^)	7.21 ± 2.23
Mean stent area luminal (mm^2^)	5.63 ± 1.83
Mean stent diameter abluminal contour (mm)	3.27 ± 0.50
Mean stent diameter mid contour (mm)	2.86 ± 0.49
Mean stent diameter endoluminal contour (mm)	2.52 ± 0.45

Data expressed as mean ± standard deviation or count (percentage). LAD = Left Anterior Descending Artery; LCX = Left Circumflex Artery; RCA = Right Coronary Artery

### Comparison of OCT and 3DStent analysis

Overall, mean stent area was 7.03 ± 2.85 mm^2^ by OCT and 9.41 ± 2.79 mm^2^, 7.21 ± 2.23 mm^2^ and 5.63 ± 1.83 mm^2^ by 3DStent at abluminal, mid and endoluminal side respectively. Mean stent diameter was of 2.93 ± 0.58 mm by OCT and 3.27 ± 0.50 mm at abluminal, 2.86 ± 0.49 mm at mid and 2.52 ± 0.45 mm at endoluminal contour by 3DStent.

**Fig. 3:**
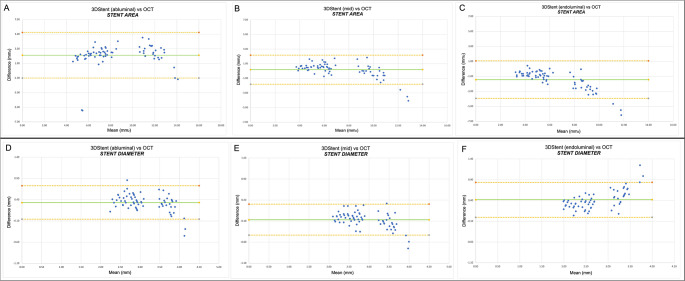
Bland Altman Plot illustrating correlation between OCT and 3DStent coronary imaging in relation to: stent area at abluminal (**A**), mid (**B**) and endoluminal (**C**) level; stent diameter at abluminal (**D**), mid (**E**) and endoluminal level (**F**). OCT = optical coherence tomography. Mean = mean value between stent area assessed by 3DStent and OCT; Difference = difference between stent assessed by 3DStent and OCT

A significant correlation was found in terms of stent area between the two imaging techniques (Exp(B): 2.64, 3.35, 4.39 for abluminal, mid and endoluminal measurement respectively; *p* < 0.001). According to Bland Altman plot the 3DStent area measured at mid side had the lowest difference as compared to OCT (mean of difference 0.19± 1.01 mm^2^, 95%CI -1.80–2.17 mm^2^) vs. abluminal (mean of difference 2.12±1.59mm^2^, 95%CI -1.00–5.23 mm^2^) and endoluminal side (mean of difference − 1.44±1.27 mm^2^, 95%CI -3.93–1.06 mm^2^) (Fig. 3).

A significant correlation was observed between the two techniques also regarding stent diameter analysis (Exp(B): 3.01, 3.18, 3.45 for abluminal, mid and endoluminal measurement respectively, *p* < 0.001) with the lowest difference for the 3DStent diameter measured at the mid side according to Bland Altman plot (mean difference −0.07±0.18 mm, 95%CI -0.43–0.30 mm) compared to abluminal and endoluminal side (mean of difference 0.33± 0.20 mm, 95%CI -0.05–0.72 mm; mean difference 0.42 mm ± 0.21 mm, 95%CI 0.00–0.83 mm, respectively. Notably, a diminished correlation was observed with larger cross-sections. (Fig. 3).

### 3DStent intra-observer and inter-observer agreement

Regarding stent area intra-observer agreement, a very strong correlation was found at abluminal (*r* = 0.85, mean of difference − 0.07 ± 1.51), mid (*r* = 0.92, mean of difference − 0.33 ± 0.92) and endoluminal (*r* = 0.91, mean of difference − 0.53 ± 0.71) side. Agreement for 3DStent measurements according to inter-observer analysis was also very strong (*r* = 0.95, mean of difference − 0.31 ± 0.64 for stent area; *r* = 0.93; mean of difference − 0.09 ± 0.17 for diameter respectively). Comparable results were observed for stent diameter.

## Discussion

To our knowledge this study is the first reporting intraprocedural use of 3DStent imaging and comparison with OCT on a cohort of patients.

The main findings of this study can be summarized as follows:

1. 3DStent appears to be a feasible and user-friendly allowing stent area and diameter assessment, with a high inter and intra-observer agreement.
2. 3DStent analysis of stent area and diameter was highly correlated with OCT analysis.
3. Specifically, stent area measured by 3DStent at the stent mid side appears to have the highest correlation and lowest variability as compared to OCT stent area.

It is well known that IVI guidance is associated with improved PCI outcomes as compared to angiography guidance; in particular, adequate stent expansion and a high MSA are key metrics in IVI-guided stent optimization [17–19]. 3DStent is a novel rotational, non-injected angiography-based imaging that features three-dimensional and multiplanar stent reconstruction, allowing for quantitative stent area and diameter assessment.

Whereas IVI guidance requires the use of additional imaging catheters, contrast administration and specific training for imaging interpretation, 3DStent may overcome these limitations. The results of this study suggest that 3DStent is a reliable and easy tool allowing for stent area evaluation, with a high reproducibility, as confirmed by excellent correlation coefficients in both intra- and inter-observer analysis. A very short learning curve for 3DStent visualization software may be considered, as looking specifically at the intra-observer agreement, we noted a slightly lower, yet very strong, coefficient for measurements performed at the abluminal side (*r* = 0.85) compared to mid and endoluminal (*r* = 0.92 and 0.91, respectively). This may be due to increased analyst expertise in optimizing image quality with better outlining of stent contours at abluminal site.

**Fig. 4:**
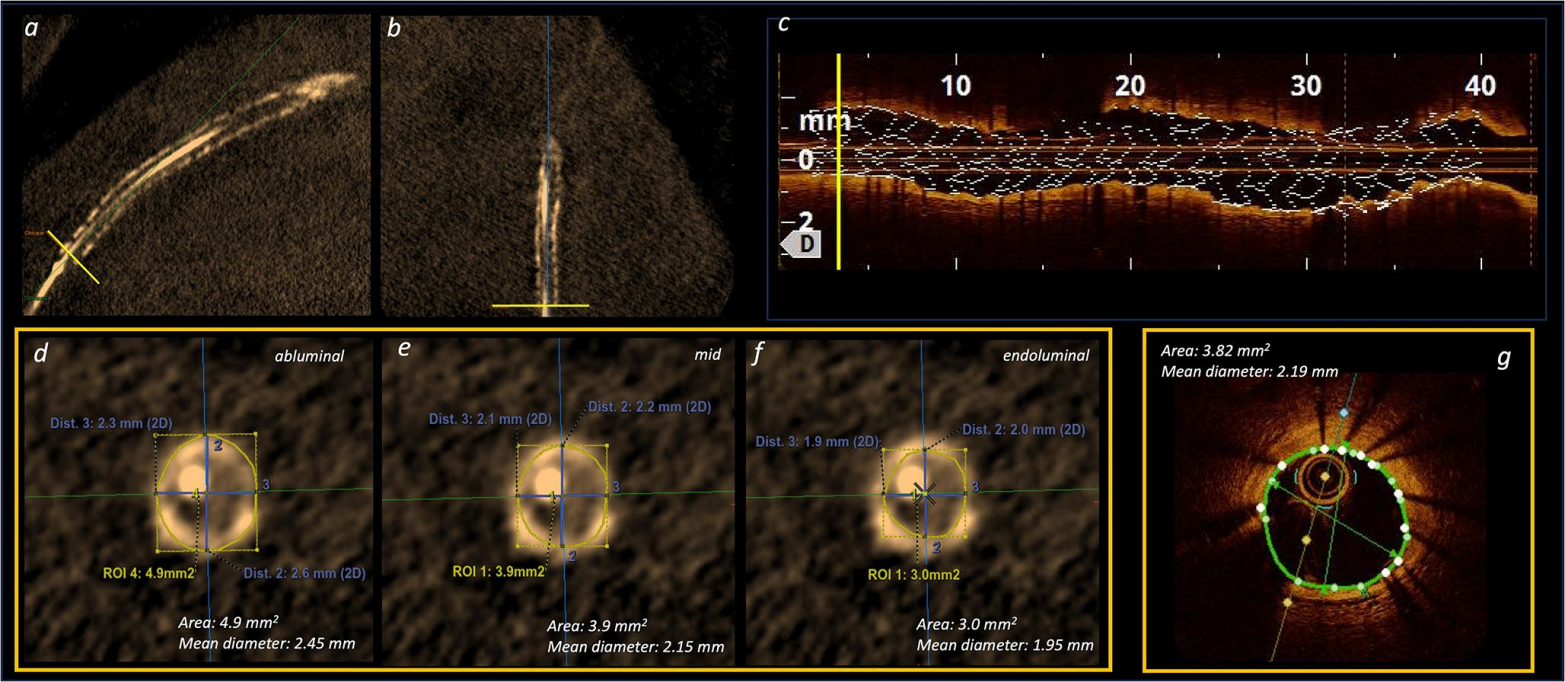
Central illustration. Comparison between 3DStent and OCT regarding cross-sectional stent area (mm^2^) and diameter (mm) performed at the same level (1.5 mm proximal from distal stent edge, yellow line). **a**-**b**: Two orthogonal longitudinal cross-sections in 3DStent reconstruction. **c**: OCT longitudinal stent reconstruction. **d–f**: 3DStent cross-sectional visualization with illustration of the 3 levels/contours of assessment of stent area and diameter: abluminal (**d**), mid (**e**) and endoluminal (**f**). **g**: OCT cross-sectional visualization and measurement of stent diameter and area. OCT = Optical Coherence Tomography; ROI: Region Of Interest

Daily use of 3DStent in evaluating post-PCI stent area does not only require high reproducible measurements but also to obtain values close to IVI. However, agreement with IVI has not been demonstrated yet, and this is the reason why our study focused on the comparison between 3DStent and OCT in terms of stent area and diameter. 3DStent software allows contouring of stent at abluminal, mid and endoluminal side at cross-section level. (Fig. 1) We have therefore compared stent area and diameter measurements using these three contours vs. OCT in order to identify the one with the highest correlation. Overall, a significant correlation was found between 3DStent and OCT, with 3DStent area measured at mid side exhibiting the highest correlation and lowest variability as compared to OCT. 3DStent area measured at abluminal and endoluminal side seem instead to overestimate and underestimate, respectively, stent area as compared to OCT. **(**Figs. 3 and 4**)** Of note, in larger measurements (area > 8 mm² or diameter > 3.5 mm), the Bland-Altman plots suggest a progressive lower correlation between 3DStent and OCT. This may indicate the need for a specific analysis focused to larger coronary vessels, such as the left main.

**Fig. 5:**
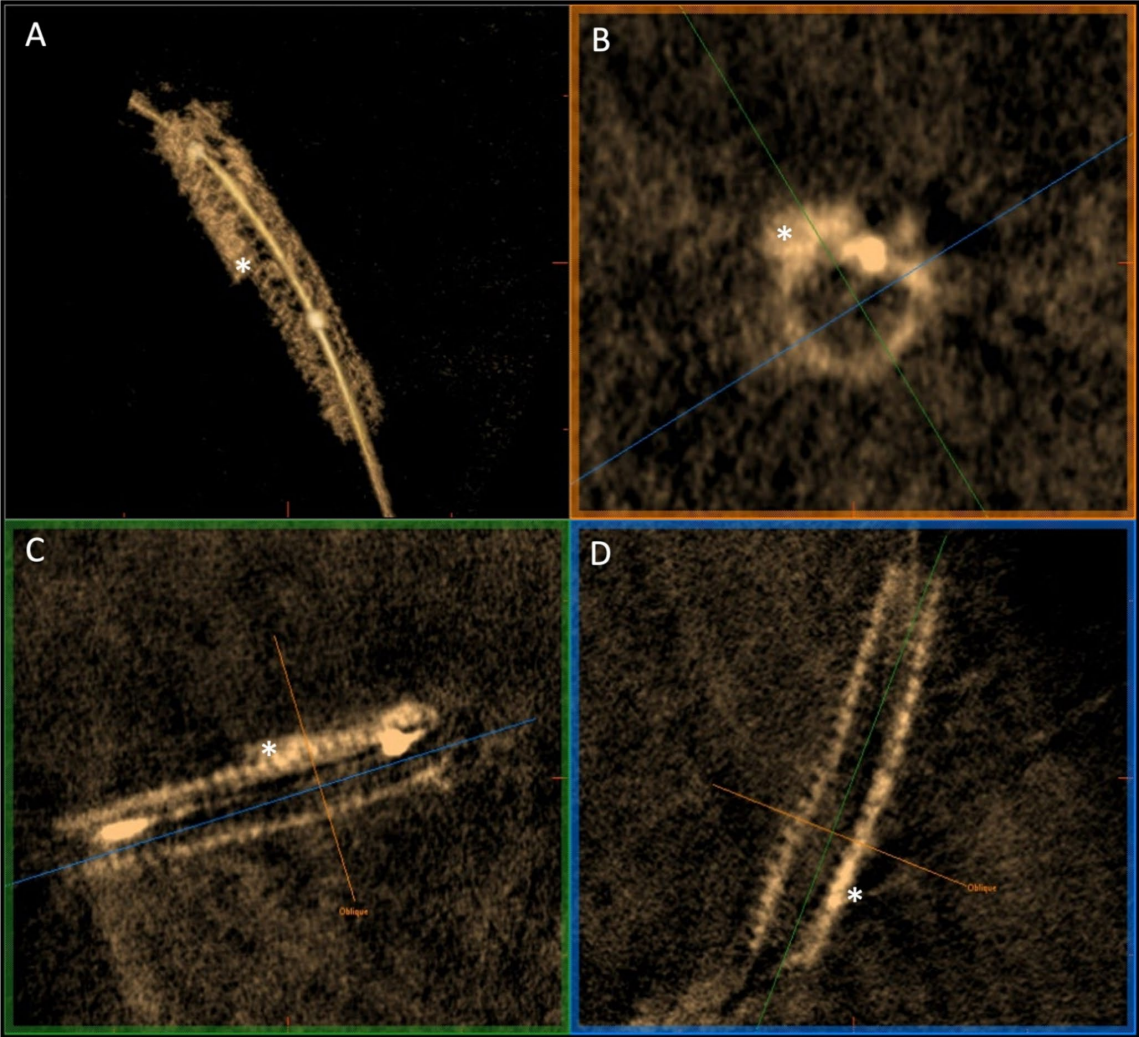
Illustration of coronary calcification (white *). **A** = 3D rendering view of 3DStent reconstruction; **B** = cross-sectional view; **C** and **D** = longitudinal orthogonal views

These findings may support future studies aiming to test 3DStent as an additional imaging modality to angiography to assess stent expansion. Comparing corresponding cross-sections in 3DStent and OCT, we have also confirmed that 3DStent technology is able to identify any radiopaque structure around the deflated balloon, such as a calcified plaque behind the stent. (Fig. 5). The role of 3DStent to quantitatively evaluate calcified lesions, either in terms of circumferential or longitudinal calcium extension or quantification of calcium thickness, should be investigated.

Further studies will be to identify patients who may benefit the most from this technology for stent optimization and to understand if 3DStent may substitute or be complementary of IVI, depending on the cases.

### Study limitations

This study has several limitations. Firstly, its retrospective design involved a small sample size from a single center. However, this technology is not yet widely available in many centers and few patients are usually enough to validate new coronary imaging software [20]. Secondly, applicability of our findings in a general population with in-stent restenosis, coronary artery bypass graft surgery, ostial lesions, chronic occlusions may be limited and should be addressed in future studies. Of note 3DStent reconstruction was not feasible in 33% of coronary lesions due to sub-optimal image quality, mainly and likely related to motion artefacts and high body mass index: this low feasibility may be related to a very early experience and it should be evaluated in a larger sample size. We report a very early real-life experience and suppose that feasibility of 3DStent may improve in the future along with the increasing expertise of physicians.

Eventually, due to its angiography-based nature, 3DStent cannot detect immediate PCI-related complications such as stent malapposition or stent edge dissection.

## Conclusion

3DStent evaluation of stent area appears to be safe, easy to perform, and reproducible. Post-PCI 3DStent cross-sectional quantitative assessment exhibits a high correlation with OCT measurements, with mid contouring of stent appearing as the one having the strongest correlation with OCT.

## Data Availability

All Data has been collected in a secure electronic registry, access allowed only to Dr. Andrea Ruberti and Dr. Salvatore Brugaletta (investigators). If required, The Authors will provide complete and accurate documentation including, but not limited to, medical records, clinical study progress records, laboratory reports, electronic case report forms, signed ICFs, device accountability records, measurements.

## Abbreviations

IVUS: Intracardiac Echocardiography
MI: Myocardial Infarction
NSTEMI: Non ST-Elevation Myocardial Infarction
OCT: Optical Coherence Tomography
STEMI: ST-Elevation Myocardial Infarction
